# A Small RNA Controls Expression of the Chitinase ChiA in *Listeria monocytogenes*

**DOI:** 10.1371/journal.pone.0019019

**Published:** 2011-04-18

**Authors:** Jesper S. Nielsen, Marianne Halberg Larsen, Eva Maria Sternkopf Lillebæk, Teresa M. Bergholz, Mie H. G. Christiansen, Kathryn J. Boor, Martin Wiedmann, Birgitte H. Kallipolitis

**Affiliations:** 1 Department of Biochemistry and Molecular Biology, University of Southern Denmark, Odense, Denmark; 2 Department of Veterinary Disease Biology, Faculty of Life Sciences, University of Copenhagen, Frederiksberg, Denmark; 3 Department of Food Science, Cornell University, Ithaca, New York, United States of America; Queen Mary University of London, United Kingdom

## Abstract

In recent years, more than 60 small RNAs (sRNAs) have been identified in the gram-positive human pathogen *Listeria monocytogenes*, but their putative roles and mechanisms of action remain largely unknown. The sRNA LhrA was recently shown to be a post-transcriptional regulator of a single gene, *lmo0850*, which encodes a small protein of unknown function. LhrA controls the translation and degradation of the *lmo0850* mRNA by an antisense mechanism, and it depends on the RNA chaperone Hfq for efficient binding to its target. In the present study, we sought to gain more insight into the functional role of LhrA in *L. monocytogenes*. To this end, we determined the effects of LhrA on global-wide gene expression. We observed that nearly 300 genes in *L. monocytogenes* are either positively or negatively affected by LhrA. Among these genes, we identified *lmo0302* and *chiA* as direct targets of LhrA, thus establishing LhrA as a multiple target regulator. *Lmo0302* encodes a hypothetical protein with no known function, whereas *chiA* encodes one of two chitinases present in *L. monocytogenes.* We show here that LhrA acts as a post-transcriptional regulator of *lmo0302* and *chiA* by interfering with ribosome recruitment, and we provide evidence that both LhrA and Hfq act to down-regulate the expression of *lmo0302* and *chiA*. Furthermore, *in vitro* binding experiments show that Hfq stimulates the base pairing of LhrA to *chiA* mRNA. Finally, we demonstrate that LhrA has a negative effect on the chitinolytic activity of *L. monocytogenes*. In marked contrast to this, we found that Hfq has a stimulating effect on the chitinolytic activity, suggesting that Hfq plays multiple roles in the complex regulatory pathways controlling the chitinases of *L. monocytogenes*.

## Introduction

Small RNAs (sRNAs) in bacteria play regulatory roles in a wide range of physiological processes, such as virulence [1], [2], iron homeostasis [3], cell envelope stress [4] and sugar metabolism [5]. Many sRNAs act as post-transcriptional regulators by base pairing to specific target mRNAs, thereby affecting their translation and/or stability [6], [7]. A single sRNA may target multiple mRNAs and typically binds to short complementary regions overlapping the ribosome-binding site and/or start codon. In these cases, sRNA-mRNA pairing prevents the ribosome from binding to the mRNA, resulting in repression of translation and rapid degradation of the mRNA. Although base-paring sRNAs are primarily known as negative regulators, some sRNAs have been found to exert a positive effect on gene expression. In these cases, sRNAs may activate translation by liberating the ribosome-binding site from an inhibitory stem-loop structure situated at the 5′ mRNA region [6], [8]. Alternatively, sRNA binding to the 5′ mRNA region may lead to processing and/or stabilization of the mRNA [9], [10].

The gram-positive human pathogen *Listeria monocytogenes* survives and multiplies in many different environments, including soil and water, food processing environments and the eukaryotic host cell cytosol [11]. Controlled expression of genes supporting the growth and survival of *L. monocytogenes* under diverse and rapidly changing conditions is likely to involve the action of regulatory sRNAs. Thus far, more than 60 sRNAs have been identified in *L. monocytogenes* [12]–[14], and a subset of these interact with the RNA chaperone Hfq [15]. In *L. monocytogenes*, Hfq contributes to stress tolerance and pathogenesis in mice [16], and similar roles for Hfq has been found in other pathogens, including *Salmonella* [17], *Vibrio cholerae* [18], *Pseudomonas aeruginosa* [19] and *Francisella tularensis* [20]. In general, Hfq has a stabilizing effect on sRNAs and facilitates the base pairing between sRNAs and their target mRNAs [21], [22]. In the gram-negative bacteria *E. coli* and *Salmonella*, all trans-acting base pairing sRNAs characterized thus far require Hfq for their function. In contrast to this, the base pairing sRNAs identified in gram-positive bacteria appear to act independently of Hfq, with the sRNA LhrA in *L. monocytogenes* being the only exception identified so far [23]. LhrA consists of 268 nucleotides and was first identified in co-immunoprecipitation experiments using antibodies directed against Hfq [15]. LhrA accumulates during growth in rich media and like most Hfq-binding base pairing sRNAs in *E. coli* and *Salmonella*, the stability of LhrA is strongly affected by Hfq. Using a computational approach in combination with *in vivo* and *in vitro* experiments, we previously showed that LhrA binds specifically to the 5′-end of the *lmo0850* mRNA, encoding a small protein of unknown function, to repress translation and stimulate degradation of *lmo0850* mRNA in an Hfq-dependent manner [23]. These findings demonstrated that LhrA acts as an Hfq-dependent anti-sense RNA, however, the functional role of LhrA in *L. monocytogenes* remained unclear.

In this work, we studied the effects of LhrA on global gene expression in *L. monocytogenes* EGD-e by a comparative microarray analysis of wild type and Δ*lhrA* mutant strains. We found that lack of *lhrA* results in the altered expression of approximately 300 genes and we further demonstrate a direct effect of LhrA on two genes: *lmo0302*, encoding a hypothetical protein with no known function, and the chitinase-encoding gene *chiA*. The chitinases ChiA and ChiB of *L. monocytogenes* catalyze the hydrolysis of the carbohydrate polymer chitin, a highly abundant carbon and nitrogen source found in the environment [24], [25]. Furthermore, *chiA* and *chiB* contribute to the pathogenesis of *L. monocytogenes* in mice, possibly through the recognition of glycoproteins or other carbohydrate moieties present in the infected host [26]. Here, we show that LhrA acts to down regulate the expression of *lmo0302* and *chiA* at the post-transcriptional level in an Hfq-dependent manner, demonstrating that LhrA is a multiple target regulator in *L. monocytogenes*.

## Results

### Dissecting the *lhrA* promoter region

**Figure 1:**
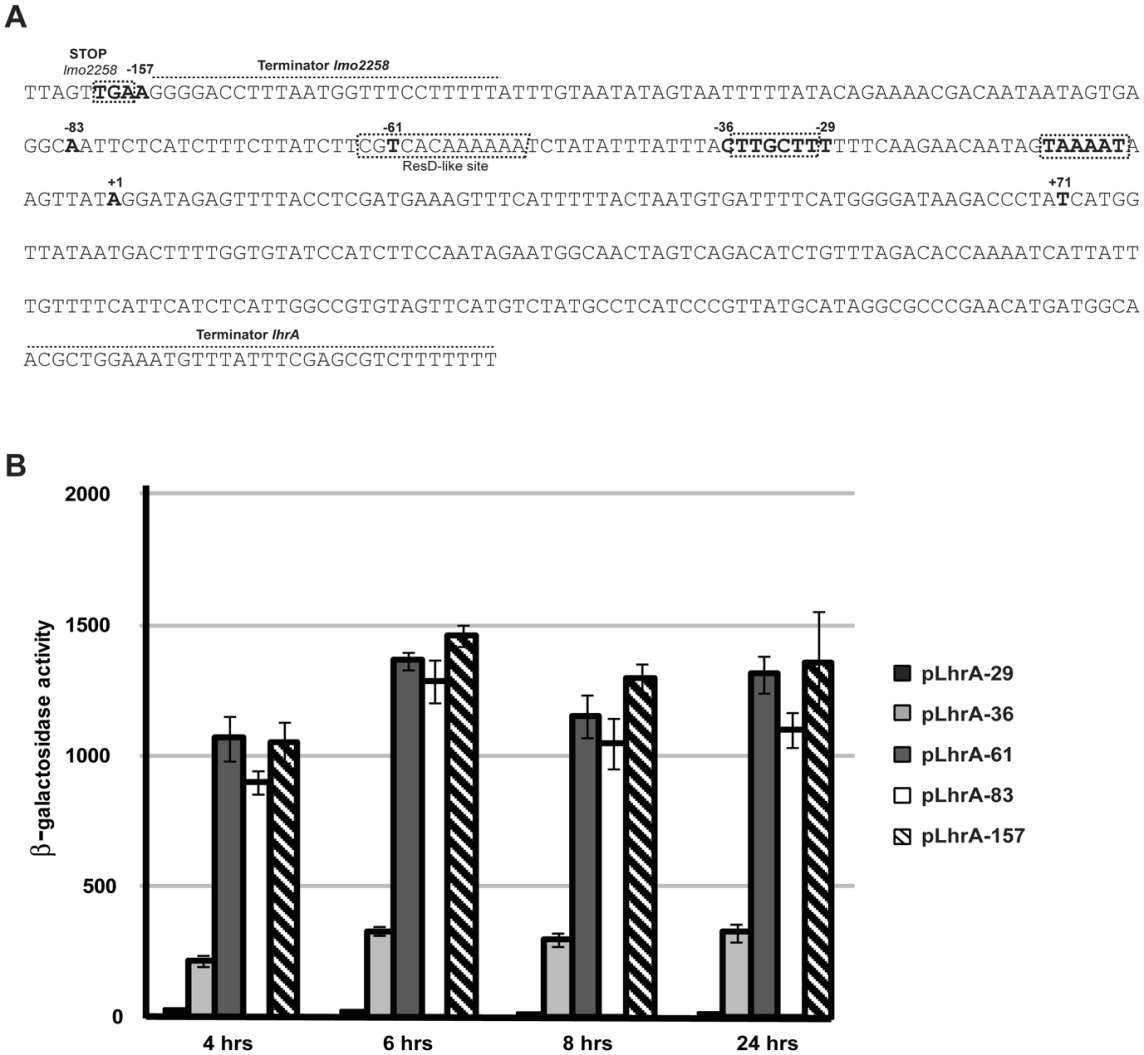
Promoter deletion analysis of *lhrA*. (A) DNA sequence showing the *lhrA* promoter region. The −35 and −10 regions are boxed, and +1 corresponds to the transcriptional start site of LhrA. The truncated *lhrA* promoter fragments fused to *lacZ* range from −29, −36, −61, −83 or −157, to +71, as indicated in the sequence. A putative binding site for ResD, identified by searching the *Bacillus subtilis* DBTBS Release 5 website (http://dbtbs.hgc.jp/) [50], is boxed. (B) Specific β-galactosidase activity of wild type cells containing transcriptional *lhrÁ-lacZ* fusions. Samples were drawn at the indicated time points through out growth. At 4 hours, the cells were in the mid-exponential growth phase; the 6 hour sample corresponds to late exponential growth phase cells, and the 8 and 24 hour samples correspond to early and late stationary phase cells, respectively. Means and standard error of the means from three independent experiments performed in duplicate are shown.

sRNAs are often highly regulated at the transcriptional level, and identification of the environmental signals and transcription factors controlling their expression may provide important clues to their biological function. Upon growth in rich media, LhrA accumulates in an Hfq-dependent manner at the entry into stationary phase, suggesting that LhrA plays a role in the transition from actively growing to resting cells ([15], [23]). To gain further insight into the transcriptional regulation of *lhrA*, we performed a *lhrA* promoter deletion analysis. To this end, truncated versions of the *lhrA* promoter were fused to a promoter-less *lacZ* gene in the transcriptional fusion vector pTCV-lac. The *lhrA* promoter fragments range from position −157, −83, −61, −36 or −29, to position +71, relative to the transcriptional start site of *lhrA* (Figure 1A). The *lhrÁ-lacZ* fusion plasmids were introduced into the *L. monocytogenes* EGD-e wild type strain and the level of specific β-galactosidase activity was determined during growth in rich medium (Figure 1B). Very high and comparable levels of β-galactosidase activity were recorded throughout growth for all constructs containing deletions of the *lhrA* promoter region down to position −61. The construct p*lhr36-lacZ*, containing the core promoter sequence of *lhrA*, displayed a 5 fold lower level of activity throughout growth (Figure 1B). Further deletion of the promoter region was expected to abolish the promoter activity, and accordingly, cells containing the construct p*lhr29-lacZ*, which lacks the −35 box, displayed background levels of activity (Figure 1B). As a control experiment, the transcription start site and RNA level of the various *lhrÁ-lacZ* transcripts were tested by primer extension analysis using a *lacZ*-specific primer. For all *lhrÁ-lacZ* constructs, only a single transcription start site was observed, corresponding to the expected start site for *lhrA*, and furthermore, the level of *lhrÁ-lacZ* transcript appeared constant throughout growth (data not shown).

Collectively, these results show that LhrA is expressed throughout growth from a highly active promoter, and that the region located between position −61 and position −36 has a stimulating effect on transcription. Within this region, we noticed a sequence motif similar to that recognized by the response regulator ResD (see Figure 1A), which is known to affect the transcription of multiple genes in *L. monocytogenes* [27]. We therefore tested the effect of ResD on transcription of p*lhrA36-lacZ* and p*lhrA61-lacZ* in a Δ*resD* mutant background. No difference in β-galactosidase activity was observed between the wild type and *resD* mutant strain (data not shown) suggesting that expression of *lhrA* is not stimulated by ResD. We also noticed the presence of an AT-rich region between position −61 and position −36 (Figure 1A). In *Escherichia coli* and *Bacillus subtilis,* AT-rich regions called UP elements, which are located upstream of the−35 region, are known to facilitate binding of the RNA polymerase to a promoter, resulting in an enhanced transcription activity [28]–[31]. The AT-rich element located between position −61 and −36 in the *lhrA* promoter region may play a similar role resulting in a highly efficient transcription of *lhrA*. No other putative regulatory elements were observed within this region.

**Figure 2:**
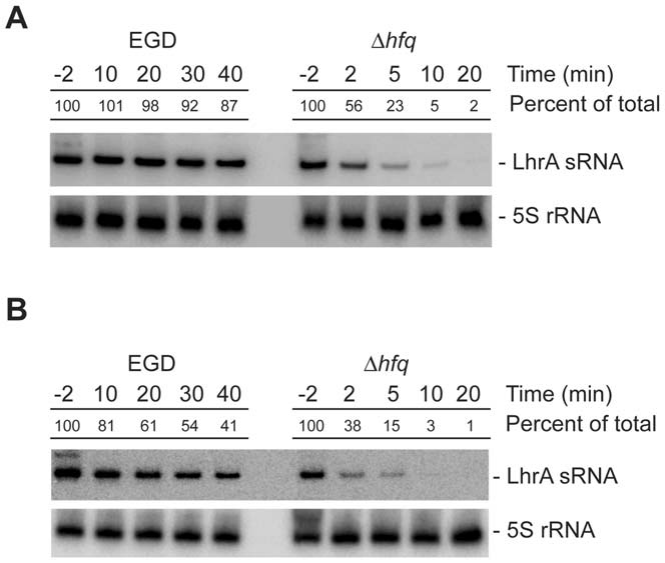
The stability of LhrA depends on the growth phase and the presence of Hfq. Northern blot analysis of LhrA and 5S rRNA (control). Total RNA samples were prepared from wild type and Δ*hfq* mutant cells grown to early stationary phase (A) or mid-exponential phase (B) and treated with rifampicin. Cells were harvested 2 minutes before (−2) and at indicated time points (in minutes) after rifampicin treatment. The experiment was repeated twice with similar results. Northern blots were quantified using ImageQuant by measuring the amount of radioactivity in each band. The numbers given are relative to the amount observed in the wild type strain at time −2 minutes. Relative expression levels were corrected with respect to the levels of 5S rRNA.

Since *lhrA* appears to be transcribed at a relatively high and constant level throughout growth, we hypothesized that the growth phase dependent accumulation of LhrA observed in Northern blots [15], [23] is likely to be the result of a post-transcriptional control mechanism. To test this hypothesis, we compared the stability of LhrA in early stationary phase (OD_600_ = 2.2) and early exponential phase cells (OD_600_ = 0.4) (Figure 2A and 2B). In both cases, LhrA appears to be extremely stable in the wild type background; however, in exponential cells, the turnover of LhrA appears to be faster (half-life approximately 30 minutes) than in early stationary cells (half-life >60 minutes). Regardless of the growth phase, the stability of LhrA is clearly facilitated by the Hfq protein. Thus, it appears that the level of LhrA is controlled mainly at the post-transcriptional level.

### LhrA affects gene expression on a global level

To learn more about the physiological role of LhrA, we performed a genome-wide transcriptome comparison of wild type and Δ*lhrA* mutant cells grown in rich growth medium until early stationary phase. Under these conditions, the transcript level of 284 genes differed significantly in the Δ*lhrA* strain in comparison to the wild type strain (≥1.5 fold; adjusted P<0.05). Of these, 191 genes were expressed at a lower level in the mutant strain (Table S1) whereas 93 genes were expressed at a higher level compared to the wild type strain (Table S2). In general, with the exception of genes encoding ribosomal proteins, no functional groups were overrepresented, suggesting that LhrA does not target any one specific group of genes under these growth conditions. The normal response to reaching stationary phase is characterized by a down-regulation of ribosome numbers in order to conserve energy [32], so a decreased level of ribosomal gene expression in the Δ*lhrA* strain may indicate that the timing of the stationary response is compromised in the absence of LhrA, although no differences in growth of the two strains could be observed, as described previously [23]. When comparing our transcriptomic data with the results from other genome-wide expression experiments in *L. monocytogenes*, we noticed an overlap with genes identified as belonging to the σ^B^ regulon [12], [33]. As indicated in Tables S1 and S2, approximately one half of the genes identified as being differentially expressed in the Δ*lhrA* mutant (131 out of 285 genes), have also been found to be affected in a Δ*sigB* strain. Strikingly, there is a high degree of inverse correlation between the effects of LhrA and σ^B^ on gene expression, suggesting a putative link between the two regulons, although it should be noted, that data on the σ^B^ regulon in *L. monocytogenes* strain 10403S was used for the comparison presented in Table S1 and S2.

To validate the microarray data, several genes were analyzed by quantitative RT-PCR (TaqMan) or Northern blotting (Figure S1 and Tables S1 and S2). In general, the results obtained by qRT-PCR and Northern blotting were consistent with the microarray data, however, some of the effects observed by microarrays could not be verified by other methods. These include the genes *glpD* and *pflA*, which based on the microarray analysis were expected to be positively affected by LhrA (Figure S1 and Table S1). Furthermore, we have previously shown that LhrA acts to destabilize the *lmo0850* mRNA [23], but to our surprise, *lmo0850* was not identified as being affected by LhrA in our microarray analysis. The reasons for these discrepancies are currently unknown.

### Identification of genes directly targeted by LhrA

**Figure 3:**
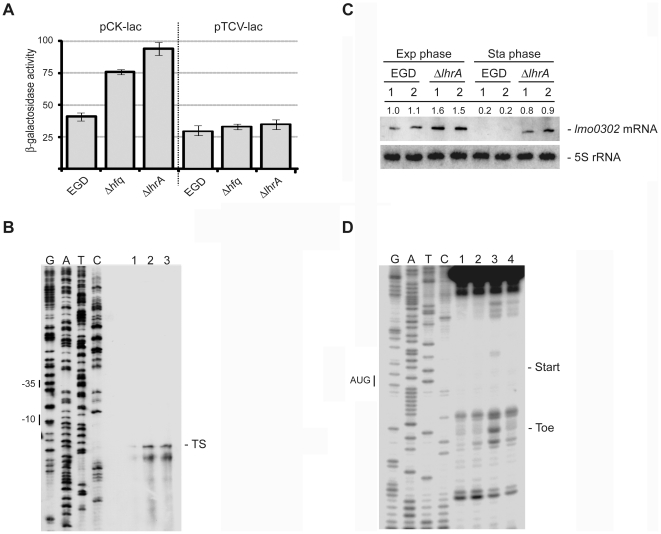
LhrA inhibits the expression of *lmo0302-lmo0303*. (A) Specific β-galactosidase activity of wild type, Δ*hfq* and Δ*lhrA* cells containing a translational (pCK-lac) or transcriptional (pTCV-lac) *lmo0302-lacZ* fusion. Cells were harvested in the mid-exponential growth phase (OD_600_ = 0.4). Means and standard error of the means from three independent experiments performed in duplicate are shown. (B) Primer extension analysis showing the transcription start site of *lmo0302* and the effect of Hfq and LhrA on *lmo0302* expression. Lane 1: wild type; lane 2: Δ*hfq*; lane 3: Δ*lhrA*. Samples were drawn from early stationary phase cells (OD_600_ = 1.0 plus 3 hours). The experiment was repeated twice with similar results. (C) Northern blot analysis showing the steady state levels of *lmo0302-lmo0303* mRNA in wild type and Δ*lhrA* mutant cells, using a probe directed against *lmo0302*. The results from two independent experiments (1 and 2) are shown. Samples were drawn in the mid-exponential growth phase and in the early stationary growth phase. The experiment was repeated twice with similar results. Northern blots were quantified using ImageQuant by measuring the amount of radioactivity in each band. Numbers given are relative to the amount found in the EGD wild type strain (exponential phase, experiment 1). Relative expression levels were corrected with respect to the levels of 5S rRNA. (D) Toeprint experiment of *lmo0302* RNA in the absence or presence of LhrA. *In vitro* transcribed *lmo0302* RNA was mixed with 30S ribosomes in the absence or presence of 10 fold excess of LhrA. Lanes 1 and 2 correspond to control reactions containing *lmo0302* RNA only, in the absence (lane 1) or presence (lane 2) of 30S ribosomes. Lane 3: In the presence of *lmo0302* RNA, 30S ribosomes and tRNA^fMet^, a specific toeprint is generated approximately 14 nucleotides downstream from the *lmo0302* start codon (AUG). Lane 4: The addition of LhrA diminishes the formation of the *lmo0302* toeprint signal. The experiment was repeated twice with similar results.

Hfq-dependent sRNAs typically act by binding to the 5′-region of target mRNAs, leading to repression of translation initiation and degradation of the mRNA. To identify genes directly controlled by LhrA, we therefore searched for potential base-pairing between the 5′-regions of mRNAs, showing at least a 2-fold increased abundance in the Δ*lhrA* mutant strain (Table S2), and the single stranded region in LhrA, shown to be important for base-pairing to the 5′-region of the *lmo0850* mRNA [23]. The three best candidates are presented in Table 1. In all three cases, LhrA is proposed to pair with the Shine Dalgarno (SD) sequence and/or translational start site (AUG), which is likely to cause translational repression. To test whether LhrA affects the expression of these candidates, in-frame translational fusions to *lacZ* were constructed in the vector pCK-*lac*. The resulting *lacZ* fusion vectors were transferred into wild type, Δ*hfq* and Δ*lhrA* mutant cells, and β-galactosidase activity was measured during growth in rich medium. The *lmo0880-lacZ* fusion was found to be expressed throughout growth at similar levels in all three strains tested (data not shown), suggesting that this gene may not be a direct target of LhrA and therefore was not considered further in the present study. In contrast, the *lmo0302-lacZ* fusion was expressed at a higher level in the Δ*lhrA* and Δ*hfq* mutant strains, suggesting that LhrA acts to down regulate the expression of *lmo0302* in an Hfq-dependent manner (see Figure 3A).

**Table 1:** Putative LhrA target genes.

Gene	Function	Fold of regulation(Δ*lhrA*/wt)	Potential base pairing^a^
*lmo0302*	Hypothetical protein of unknown function	2.5	*lmo0302* mRNA 5′-CAUAG-GAGAUGAAUAGAUGAUGAAAAAAA-3′ :|: ||||||||| || :|::|| || LhrA 3′-CGGUUACUCUACUUA-CUUUUGUUUAUUAC-5′
*lmo0880*	Putative cell wall associated protein precursor (LPXTG motif)	2.0	*lmo0880* mRNA 5′-UUUUAA-GGGG-GAAUG-AAACAAAAUGAA-3′ ::|| |:|| ||||| ||||||| : LhrA 3′-CCGGUUACUCUACUUACUUUUGUUUAUUAC-5′
*chiA* (*lmo1883*)	Chitinase ChiA	3.3	*chiA* mRNA 5′-AGUAGAUGAAUGGAAAACAAGUAAUGGUUGG-3′ |||||||| ||||||||||||||:|| LhrA 3′-UACUCUACUUA-CUUUUGUUUAUUACUAAAA-5′

a Start codons (AUG) are indicated in bold. In the predicted duplex formed by *chiA* mRNA and LhrA, the underlined nucleotides in LhrA were substituted from UGUU to ACAA in the mutant version LhrA-Mut3*.

According to the genome sequence of *L. monocytogenes* EGD-e, *lmo0302* is the first gene in an operon consisting of two genes, *lmo302* and *lmo0303*. The *lmo0302* gene is predicted to encode a hypothetical protein of 94 amino acids, whereas *lmo0303* encodes a putatively secreted, lysine rich protein of 184 amino acids. The results of the microarray analysis suggested that both *lmo0302* and *lmo0303* are negatively affected by LhrA (2.5 fold and 2.2 fold, respectively, see Table S2). To map the 5′-end of the putative *lmo0302-lmo0303* transcript, we performed a primer extension analysis, using total RNA purified from early stationary phase cells (Figure 3B). For the Δ*lhrA* and Δ*hfq* mutant strains, we observed two bands corresponding to putative 5′-ends mapping to position −38 and –34 relative to the translation start site for *lmo0302*. Putative −10 and −35 sequences are located 8 bp upstream of position −38 (Figure 3B) suggesting that transcription of *lmo0302* starts at this site. Furthermore, the primer extension analysis clearly showed that the level of transcription is higher in the Δ*lhrA* and Δ*hfq* mutant strains, relative to the wild type.

To study the expression of the *lmo0303* gene, which is expected to be co-transcribed with *lmo0302*, we performed a Northern blot experiment using a radio-labeled probe directed against *lmo0303* RNA. We observed a single transcript of around 1000 nucleotides, which could be expected to encompass both genes (Figure 3C). Furthermore, we note that the level of the *lmo0302-lmo0303* transcript was clearly higher in the Δ*lhrA* mutant relative to the wild type strain in both exponential phase and stationary phase cells. Identical results were obtained when using a probe directed against *lmo0302* mRNA (data not shown). From these results we conclude that LhrA acts to down-regulate *lmo0302* and *lmo0303* at the RNA level. To investigate whether this regulatory effect occurs at the level of transcription initiation, the *lmo0302* promoter region was fused to *lacZ* in the transcriptional fusion vector pTCV-lac. The resulting plasmid was introduced into the wild type, Δ*lhrA* and Δ*hfq* mutant strains, and the β-galactosidase activity was measured throughout growth in rich medium. The three strains displayed no difference in β-galactosidase activity (Figure 3A), suggesting that the regulation of *lmo0302* and *lmo0303* by LhrA and Hfq indeed occurs at the post-transcriptional level.

According to the predicted interaction between LhrA and *lmo0302* mRNA, LhrA binds to a region overlapping the SD region as well as the start codon (Table 1). To investigate if LhrA inhibits the formation of a translation initiation complex on the *lmo0302* mRNA, we performed a toeprint experiment (Figure 3D). An *in vitro* transcribed *lmo0302* mRNA fragment was incubated with 30S ribosomes in the absence or presence of uncharged tRNA^fMet^ followed by primer extension. In the presence of tRNA^fMet^, specific binding of 30S ribosomes to the *lmo0302* mRNA generates a toeprint signal downstream from the start codon (Figure 3D, lane 3). When LhrA was added, the toeprint signal was clearly diminished (Figure 3D, lane 4), demonstrating that LhrA efficiently prevents the formation of a translation initiation complex at the *lmo0302* mRNA.

**Figure 4:**
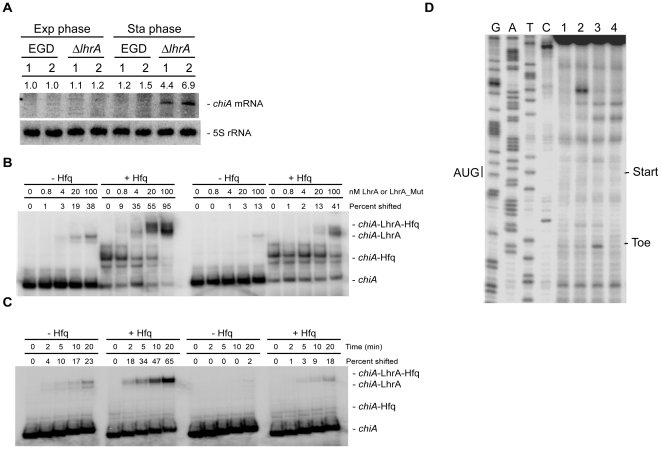
LhrA specifically targets the *chiA* mRNA. (A) Northern blot analysis of *chiA* expression in wild type and Δ*lhrA* mutant cells grown in BHI medium. The results from two independent experiments (1 and 2) are shown. Samples were drawn in the mid-exponential growth phase and in the early stationary growth phase. Northern blots were quantified using ImageQuant by measuring the amount of radioactivity in each band. Numbers given are relative to the amount found in the EGD wild type strain (exponential phase, experiment 1). Relative expression levels were corrected with respect to the levels of 5S rRNA. (B) *In vitro* binding assay of LhrA and *chiA* RNA in the absence (−) or presence (+) of Hfq. In all samples, end-labelled *in vitro* transcribed *chiA* RNA was used, and *in vitro* transcribed LhrA (left part) or LhrA-Mut3* (right part) was added to the indicated final concentrations. The experiment was repeated twice with similar results. Quantification of the gelshifts was carried out using ImageQuant by measuring the amount of RNA that had shifted in each lane. The numbers presented in the figure are relative to the total amount of unshifted RNA found in the first lane, from the left. (C) Time course experiment showing the association of *chiA* RNA with LhrA (left part) or LhrA-Mut3* (right part). The experiments were carried out with end-labelled *in vitro* transcribed *chiA* RNA mixed with *in vitro* transcribed LhrA or LhrA-Mut3*, in the absence (−) or presence (+) of Hfq. The samples were incubated at 37°C for 0, 2, 5, 10 or 20 minutes and then loaded onto a gel. The experiment was repeated twice with similar results. Quantification of the bands was carried out as described for 4B. (D) Toeprint experiment of *chiA* RNA in the absence or presence of LhrA. Lanes 1 and 2 correspond to control reactions containing *chiA* RNA only, in the absence (lane 1) or presence (lane 2) of 30S ribosomes. Lane 3: In the presence of *chiA* RNA, 30S ribosomes and tRNA^fMet^, a specific toeprint is generated approximately 18 nucleotides downstream from the *chiA* start codon (AUG). Lane 4: The addition of LhrA diminishes the formation of the *chiA* toeprint signal. The experiment was repeated twice with similar results.

The third candidate predicted to base pair with LhrA, the *chiA* mRNA, was identified previously in a computational search for putative LhrA target genes [23]. In our previous study, we constructed a translational fusion of *chiA* (*lmo1883*) to *lacZ* in pCK-lac, but no detectable β-galactosidase activity was recorded, and the *chiA* gene was not investigated any further [23]. However, according to the microarray analysis, the expression of *chiA* is 3.3 fold higher in a Δ*lhrA* mutant strain relative to the wild type, suggesting that LhrA has a negative effect on the level of *chiA* mRNA. To investigate this in more detail, we performed a Northern blot analysis to evaluate the expression of *chiA* mRNA in wild type and Δ*lhrA* cells during growth in rich medium. As shown in Figure 4A, the expression of *chiA* is very low in the wild type strain, irrespective of the growth phase tested. Likewise, the expression of *chiA* in the Δ*lhrA* mutant strain is minimal in exponentially growing cells, however, at the entry into stationary phase, the level of *chiA* mRNA increases (Figure 4A). Thus, LhrA indeed has a negative affect the level of *chiA* mRNA.

LhrA shows extensive complementarity to the translation initiation region the 5′-end of *chiA* mRNA (see Table 1). To test whether LhrA binds to this region in the *chiA* mRNA, we performed gel mobility shift assays (Figure 4B). In these experiments, a 5′-end-labelled *in vitro*-transcribed *chiA* RNA was mixed with different concentrations of wild type LhrA or mutant LhrA-Mut3* carrying four nucleotide substitutions within the region predicted to interact with *chiA* RNA (see Table 1 and [23] for details). The binding experiments were performed in the absence or presence of Hfq. In the absence of Hfq, less than half of the *chiA* RNA had shifted at the highest concentration of wild type LhrA, whereas in the presence of Hfq, almost all of the *chiA* RNA had shifted (Figure 4B, left panel). When adding LhrA-Mut3*, RNA duplex formation was clearly diminished, both in the absence and presence of Hfq (Figure 4B, right panel). These results show that LhrA interacts with the 5′-end of the *chiA* mRNA, and that Hfq promotes the formation of an LhrA-*chiA* RNA duplex. To further investigate this issue, we conducted a time course experiment in which a 5′-end-labelled *chiA* RNA fragment was mixed with 5-fold excess LhrA or LhrA-Mut3*, in the absence or presence of Hfq (Figure 4C). The results clearly show that Hfq stimulates the rate of association between LhrA and *chiA* RNA, and that substitutions in LhrA predicted to disrupt this interaction diminishes RNA duplex formation.

Since LhrA binds to a region overlapping the start codon on the *chiA* mRNA (see Table 1), we performed a toeprint experiment to investigate the effect of LhrA on the formation of a translation initiation complex. In the presence of both 30S and tRNA^fMet^ a distinct toeprint signal was observed downstream from the start codon (Figure 4D, lane 3). The formation of the toeprint was strongly inhibited by the addition of LhrA RNA (Figure 4D, lane 4), showing that binding of LhrA to *chiA* mRNA efficiently blocks the formation of a translation initiation complex.

### LhrA and Hfq affect the chitinolytic activity of *L. monocytogenes*

**Figure 5:**
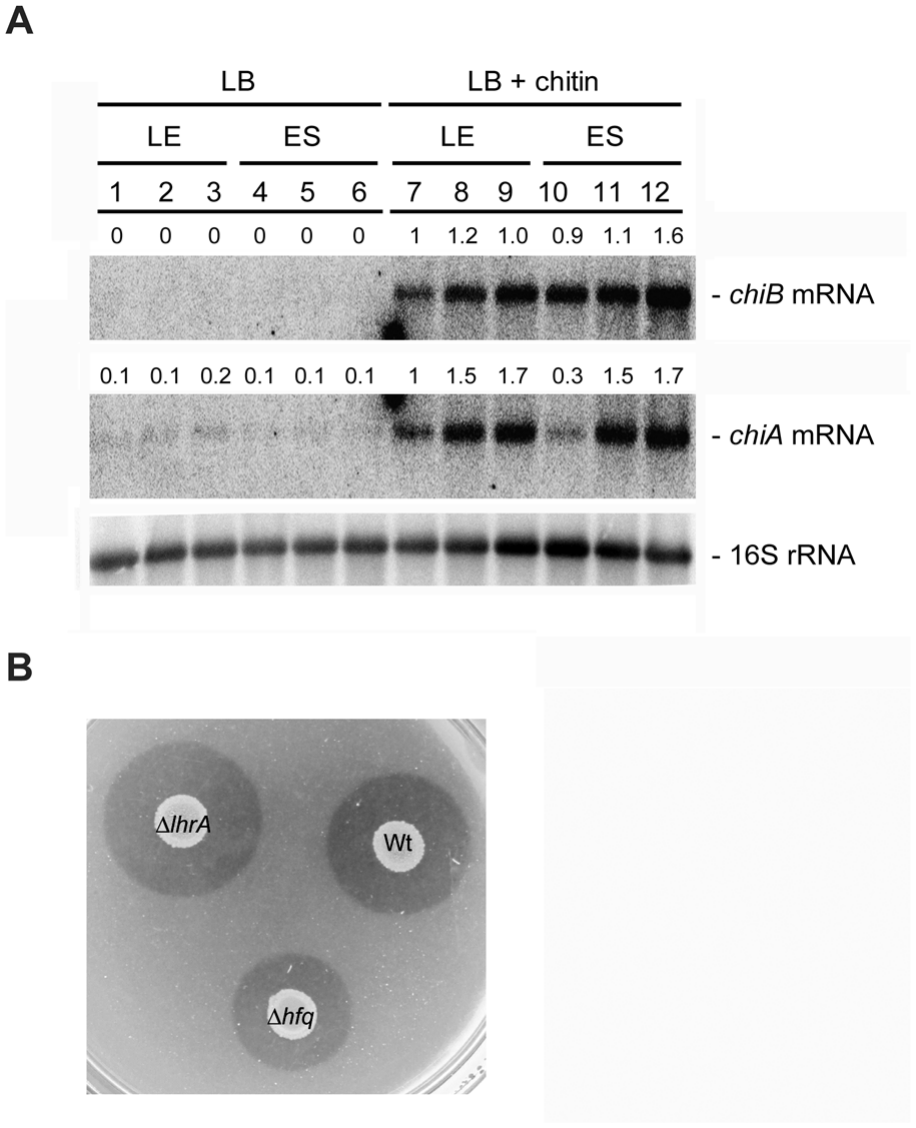
The effect of LhrA and Hfq on *chiA* and *chiB*. (A) Northern blot showing the levels of *chiA* and *chiB* mRNA in wild type (lanes 1, 4, 7, 10), Δ*lhrA* mutant (lanes 2, 5, 8, 11) and Δ*hfq* mutant cells (lanes 3, 6, 9, 12) grown in LB medium (lanes 1–6) or LB medium supplemented with chitin (lanes 7–12). Cells were harvested in the late exponential growth phase (lanes 1–3 and 7–9) or early stationary growth phase (lanes 4–6 and 10–12). The experiment was repeated twice with similar results. As loading control, we probed for 16S rRNA. Northern blots were quantified using ImageQuant by measuring the amount of radioactivity in each band. Numbers given are relative to the amount found in the EGD wild type strain grown to exponential phase in the presence of chitin (lane 7). Relative expression levels were corrected with respect to the levels of 16S rRNA. (B) Comparison of the chitinolytic activity of wild type, Δ*lhrA* and Δ*hfq* mutant cells on agar plates containing chitin. Similar results were observed in ten independent experiments.

*L. monocytogenes* contains two genes encoding chitinases, *chiA* and *chiB*, and a single gene (*lmo2467*) encoding a putative chitin binding protein [24]. The chitinase activity of *L. monocytogenes* EGD-e has been shown to depend on both *chiA* and *chiB*, and both chitinases contribute to growth in the livers and spleens of mice, with *chiA* playing the most significant role in *L. monocytogenes* virulence [26]. Expression of the chitinase genes is induced in the presence of chitin [34]. In order to study the effect of LhrA on *chiA* and *chiB* expression under conditions known to stimulate chitinase activity, we performed a Northern blot on RNA isolated from late exponential and early stationary phase cells grown in LB medium containing chitin (Figure 5A, lanes 7–12). In agreement with the results presented previously [34] the *chiA* and *chiB* mRNAs are readily detected in the wild type strain in the presence of chitin. We note that *chiA* expression is higher in the Δ*lhrA* and Δ*hfq* mutant strains in comparison to the wild type strain, in particular in the early stationary phase cells (Figure 5A, lanes 7–9, lower band), whereas the effect of Hfq and LhrA on *chiB* expression appears to be minimal (upper band). These results demonstrate that LhrA and Hfq act to repress the expression of *chiA*, also in the presence of the substrate of the chitinases.

In order to study the contribution of LhrA and Hfq to the chitinolytic activity of *L. monocytogenes*, bacterial suspensions of the wild type, Δ*lhrA* and Δ*hfq* mutant strains were spotted on LB agar plates supplemented with chitin (Figure 5B). We observed that the chitinolytic activity of the Δ*lhrA* mutant strain is slightly higher compared to that of the wild type strain. The average zone size of the wild type strain (1.5 mm, SD = 0.4) was found to be significantly smaller (P<0.01) compared to the average zone size of the Δ*lhrA* mutant strain (1.9 mm, SD = 0.4). This result shows that the regulatory effect of LhrA on *chiA* indeed serves to decrease the chitinolytic activity of *L. monocytogenes*. Surprisingly, we found that the clearing zone for the Δ*hfq* mutant was markedly reduced when compared to the wild type strain (Figure 5B). Since Hfq acts to down-regulate the *chiA* levels in the presence of chitin (Figure 5A) we expected Hfq to display a negative effect on the chitinolytic activity to approximately the same extent as LhrA. In contrast to our expectations, the average zone diameter of the Δ*hfq* mutant (0.9 mm, SD = 0.3) was significantly smaller (P<0.01) compared to that of the wild type strain. Thus, it appears that Hfq has a stimulating effect on the chitinolytic activity of *L. monocytogenes*, which is not reflected at the RNA level.

## Discussion

In the present work we studied the role of the Hfq-binding sRNA LhrA in *L. monocytogenes*. We demonstrate that LhrA is expressed throughout growth in rich medium from a highly active promoter, and that LhrA is more stable in stationary phase cells in comparison to exponentially growing cells. Furthermore, we show that LhrA affects the expression of approximately 300 genes in early stationary phase cells (≥1.5 fold; adjusted P<0.05), and we provide evidence that two additional genes, *lmo0302* and *chiA*, are a direct target for LhrA, extending the regulatory capacity of LhrA to multiple target genes.

To identify genes affected by LhrA, we compared the total gene expression of a Δ*lhrA* mutant and wild type strain by microarray analysis. These types of analyses are often complicated by secondary effects resulting from regulation of primary targets. Furthermore, deletion of *lhrA*, which is one of the most abundant RNAs in stationary phase cells [12], may have indirect effects on other Hfq-dependent processes in *L. monocytogenes*. We also note that several genes encoding regulatory proteins were among those differentially expressed in the Δ*lhrA* strain (Table S1 and Table S2), and that some of the observed differences could potentially be due to differences in the regulation of entry into stationary phase between the strains tested. Thus, the LhrA-mediated regulation of 284 genes in *L. monocytogenes* is most likely the result of both direct and indirect effects. In order to find genes directly targeted by LhrA, we searched the 5′-region of putative mRNAs encoded by the genes down-regulated at least 2 fold by LhrA for potential RNA-duplex formation. By this strategy, we expected to find mRNAs that interact with LhrA, leading to repression of translation initiation and degradation of the mRNA. Further analyses of the top three candidates confirmed that LhrA down-regulates the expression of the *lmo0302*-*lmo0303* operon and of *chiA*. Using *in vivo* and *in vitro* analyses, we showed that LhrA acts to prevent the formation of a translation initiation complex at the 5′-end of the *lmo030*2-*lmo0303* and *chiA* mRNAs, resulting in a decrease in the mRNA levels. We observed that the expression of these genes is affected by the RNA chaperone Hfq as well, and that Hfq stimulates the base-pairing between LhrA and *chiA* mRNA.

Some Hfq-binding sRNAs act by binding to the coding region of mRNAs, as exemplified by MicC in *Salmonella* [35], or by binding to the 5′-region more upstream from the translational start site, resulting in activation of translation, as shown for DsrA in *E. coli* [36], [37]. We therefore searched within the coding region as well as the far 5′-upstream regions of genes affected at least 2-fold by LhrA for potential base-pairing with LhrA, but no obvious targets were identified. Curiously, we note that approximately half of the genes identified in the microarray analysis were found in other studies to belong to the σ^B^ regulon, suggesting a regulatory link between LhrA and the alternative stress sigma factor σ^B^. In *L. monocytogenes*, σ^B^ plays an important role in stress tolerance and virulence, and several sRNA-encoding genes are known to depend on σ^B^ for their expression, including *sbrA* [14] and *sbrE* (*rli47*) [12], [13], but the level of LhrA is not affected by σ^B^ (our unpublished data). Further studies will be needed in order to clarify the extensive regulatory networks involving σ^B^ and sRNAs in *L. monocytogenes*.

Our studies revealed a role for LhrA in controlling the chitinase ChiA in *L. monocytogenes*. Chitin is a polymer of β-1, 4-N-acetyl-glucosamine (GlcNAc) and the second most abundant polysaccharide in nature [38]. Chitin is primarily degraded by chitinases, which are produced by a wide range of living organisms, including bacteria. In general, bacterial chitinases are associated with carbon and nitrogen acquisition, however in some pathogens, chitinases and chitin-binding proteins have been linked to bacterial pathogenesis, although chitin is not present in mammalian hosts. In *Vibrio cholerae*, the chitin-binding protein GbpA interacts with intestinal mucin and contributes to bacterial colonization of the intestine [39], [40], and in *Legionella pneumophila*, a chitinase was shown to promote bacterial persistence in the lung [41]. In *L. monocytogenes*, the chitin-binding protein Lmo2467 and the chitinases ChiA and ChiB contribute to pathogenesis in mice, but appeared not to influence bacterial invasion or replication within selected mammalian cell lines [26]. The expression of *chiA* and *chiB* is induced by the presence of chitin and depends on at least two regulatory proteins: The central virulence regulator PrfA and the alternative stress sigma factor σ^B^ [34]. Furthermore, glucose has a negative effect on *chiA* and *chiB* expression, suggesting that the chitinases are subject to catabolite repression [34]. We show that LhrA acts as a post-transcriptional regulator of *chiA* thus adding another layer of complexity to the gene regulatory networks controlling the expression of chitinases in *L. moncoytogenes*. We have shown that LhrA acts to down-regulate the expression of *chiA* by an antisense-mechanism. However, the environmental signal and molecular mechanism leading to alleviation of LhrA repression, remains to be determined. We speculate that under specific growth conditions, the LhrA level may decrease via repression of transcription of *lhrA*, and/or by removal of cellular LhrA by degradation. Interestingly, the utilization of chito-sugars by *E. coli* was recently shown to involve a small Hfq-dependent sRNA named MicM [42], and a similar sRNA, ChiX, was characterized in *Salmonella* [43]. Under normal growth conditions, MicM down-regulates its target gene *ybfM*, encoding a chito-sugar porin. In the presence of chitobiose, an RNA trap is produced from the chitobiose operon. The RNA trap binds to MicM, leading to MicM degradation and alleviation of repression of the YbfM chito-sugar porin [42], [43]. It is tempting to speculate that a similar mechanism could apply to the LhrA-*chiA* regulatory case, but if so, what is the potential signal leading to alleviation of LhrA repression? In the presence of chitin, the expression of *chiA* is clearly down-regulated by LhrA, so the putative signal does not appear to be linked directly to the substrate (chitin) or its degradation products. Although both ChiA and ChiB contribute to the chitinolytic activity of *L. monocytogenes*, the full range of their potential substrates remains to be determined, including the identification of host-related targets. Since only *chiA*, and not *chiB*, is targeted by LhrA, we speculate that LhrA-regulation could be linked to a ChiA-specific substrate in the external environment, or possibly during infection, which would require differential regulation of the two genes. This hypothesis is supported by recent findings showing that a *Salmonella* Typhimurium chitinase shows activity towards a N-acetyllactosamine-conjugate which is a model substrate to LAcNAc terminating glycoproteins and glycolipids on vertebrate cells [44]. Future work will focus on defining the mechanism leading to de-repression of LhrA-regulated genes.

The role of LhrA as an antisense regulator of the expression of *lmo0850* [26], *lmo0302-lmo0303* and *chiA* is closely linked to the RNA chaperone Hfq. The stability and function of LhrA depends on Hfq and the levels of all three target mRNAs are diminished in the presence of Hfq. The formation of an RNA duplex between LhrA and target mRNA is clearly stimulated by the Hfq protein, as shown for *lmo0850* [23] and *chiA* mRNA (this study). We were therefore surprised to find that the Δ*hfq* mutant was less chitinolytic in comparison to wild type strain. Importantly, this stimulatory effect of Hfq on the chitinolytic activity of *L. monocytogenes* was not reflected at mRNA level of *chiA* or *chiB*. This result points to a more complex role of Hfq in *L. monocytogenes* and suggests that Hfq exerts a stimulating effect on the protein level, activity and/or secretion of the chitinases by a mechanism that may involve the action of other Hfq-dependent sRNAs in *L. monocytogenes*.

## Materials and Methods

### Bacterials strains and growth media

*Listeria monocytogenes* EGD-e seroptype 1/2a was used as the wild type strain; construction of the isogenic mutant derivatives Δ*hfq* and Δ*lhrA* were described in previous work [16], [23]. The Δ*resD* mutant strain was described previously [27]. All *L. monocytogenes* strains were grown in either Brain Heart Infusion media (BHI, Oxoid), at 37°C, or Luria broth (LB, Oxoid), at 30°C. The effect of chitin was examined by supplementing LB with acid-hydrolyzed chitin (2.5 g/L) (catalog nr. C9213; Sigma-Aldrich). Acid-hydrolyzed/colloidal chitin was prepared as described previously [34]. When appropriate, cultures were supplemented with Kanamycin (50 µg/ml). For cloning purposes, *E. coli* TOP10 (Invitrogen) grown in LB medium was used.

### Construction of lacZ fusions and β-galactosidase assays

For analysis of the *lhrA* promoter activity, DNA fragments corresponding to various lengths of regions upstream of *lhrA* were constructed by PCR using different LhrA forward primers in combination with the reverse primer sRNA1-13, listed in Table S3. The resulting PCR fragments were digested with EcoRI and BamHI and ligated into the low-copy number promoter-less *lacZ* transcriptional fusion vector pTCV-lac [45]. For the construction of in-frame translational *lacZ* fusions, DNA fragments containing 5′-regions of *lmo0880* or *lmo0302* were amplified by PCR using the primers listed in Table S3. The resulting PCR fragments were digested with EcoRI and BamHI and ligated into pCK-lac, a derivative of pTCV-lac containing a *lacZ* gene without a Shine-Dalgarno sequence or start codon allowing for translational analysis of the gene in question. For construction of a transcriptional fusion between *lmo0302* and *lacZ*, the *lmo0302* PCR fragment digested with EcoRI and BamHI was ligated into pTCV-lac. β-galactosidase assay was carried out as described previously [16].

### RNA techniques

RNA used for microarray or TaqMan RT-PCR was purified using the RNeasy mini or midi kit from Qiagen as described by the manufacturer. Cells grown to early-stationary phase were first treated with RNA protect as instructed by the manufacturer (Qiagen) and subsequently disrupted by sonication on ice (3×30 seconds, each round followed by a 30 second pause). For primer extension analysis and Northern blotting experiments, total RNA was extracted from *L. monocytogenes* using TRI reagent (MRCGENE). Cells were disrupted using the FastPrep instrument and RNA was purified as described previously [23]. The integrity of the RNA was confirmed by agarose gel electrophoresis and the concentration and purity was determined on a NanoDrop 2000.

Northern blotting and primer extension analysis on total RNA purified from cells grown in BHI medium was performed as previously described [23]. The primers used as probes for Northern blotting on LhrA, 5S RNA, *lmo0302*, *lmo0303* and *chiA*, and primers used for primer extension analysis of *lhrÁ-lacZ*, *lmo0302* and *chiA*, are listed in Table S3. Northern blotting analysis of *chiA* and *chiB* on total RNA purified from cells grown in LB medium, with or without chitin, was performed at described previously [34].

For gel shift experiments, the template for *in vitro* transcription of *chiA* RNA was prepared by PCR using the primers listed in Table S3. The 5′-end corresponds to the putative transcription start site from a σ^B^ dependent promoter, located 50 base pairs upstream of the start codon [33]. Templates for *in vitro* transcription of LhrA and LhrA-Mut3* were prepared as described previously [23]. In each case, the 5′-end primer contains a T7-RNA Polymerase binding site for subsequent *in vitro* transcription. *In vitro* transcribed RNA was prepared using the MegaScript kit from Ambion as described by the manufacturer. Following transcription, the RNA was separated on a denaturing polyacrylamide gel and the largest transcript (identified by UV shadowing) was excised from the gel and subsequently purified by electro-elution followed by phenol-chloroform extraction. RNA to be used for 5′-end labeling was dephosphorylated using the KinaseMax kit from Ambion as described by the manufacturer. The purity and concentration of *in vitro* transcribed RNA was determined using a NanoDrop 2000. Gelshifts were conducted as previously described [23]. Briefly, 40 fmol 5′-end labeled *chiA* RNA was incubated in a total of 10 µl without or with 0.8, 4, 20 or 100 nM unlabelled LhrA or LhrA-Mut3* in the absence or presence of 2.5 µM Hfq and 10 µg of non-specific tRNA. The samples were incubated 20 min at 37°C followed by 10 min on ice and subsequently separated on a 5% non-denaturing polyacrylamide gel at 4°C with the current running. For time-course experiments, 40 fmol 5′-end labeled *chiA* RNA was mixed with 20 nM LhrA in the presence or absence of 2.5 µM Hfq and incubated at 37°C for 0, 2, 5, 10 or 20 minutes. The samples were then loaded onto a 5% non-denaturing polyacrylamide gel at 4°C with the current running.

For toeprinting experiments, *in vitro* transcribed *lmo0302* RNA and *chiA* RNA was prepared using the primers listed in Table S3. Toeprinting experiments were performed as described in [23] using 0.35 µM *lmo0302* RNA or 0.05 µM *chiA* RNA; 10 fold excess of *in vitro* transcribed LhrA (prepared as described for gel shift experiments), relative to *lmo0302* or *chiA* RNA, and 0.4 pmol of 5′-end labelled *lmo0302* or *chiA* primer (see Table S3).

Quantitative RT-PCR was essentially performed as described previously [46]. Briefly, TaqMan primers and probes (Table S3) were designed using Primer Express 2.0 software (Applied Biosystems). qRT-PCR was performed using TaqMan one-step RT-PCR master mix reagent, Multiscribe RT, and an ABI Prism 7000 sequence detection system (Applied Biosystems). Each qRT-PCR experiment was run in triplicate. The housekeeping genes *rpoB* and *gap* were used for normalization of absolute transcript levels. Data analysis was conducted with ABI Prism 7000 SDS software. Significant differences in RNA levels were determined by ANOVA as described previously [46].

### cDNA labeling and microarray hybridization

RNA was extracted from wild type and Δ*lhrA* cells grown in BHI medium at 37°C to early stationary phase (OD_600_ = 1.0+3 hours) as described above. The experiment was conducted with four biological replicates which were compared in pairs on four microarray slides. cDNA from each strain was labeled twice with Cy3 and twice with Cy5 to minimize any bias. cDNA labeling was performed as previously described [46]. Briefly, cDNA synthesis and labeling of total RNA were performed using the SuperScript Plus indirect cDNA labeling system for DNA microarrays (Invitrogen). 10 µg total RNA was mixed with 5 µg random hexamers and incubated for 10 minutes at 70°C, with a subsequent chill on ice for at least 5 minutes. Superscript III RT, amino-modified deoxynucleoside triphophates, dithiothreitol, RNaseOUT, and buffer was then added and the reaction mix incubated at 42°C for 17 hours. RNA was hydrolyzed by the addition of 10 µl 1 M NaOH and 10 µl 0.5 M EDTA, followed by incubation at 65°C for 15 minutes. The mixture was neutralized with 10 µl 1 M HCl and cDNA purified using the Qiagen PCR purification kit. Labeling reactions with Alexa Fluor 555 or Alexa Fluor 647 fluorescent dyes were performed for 2 h at room temperature. Differentially labeled cDNAs from the two strains to be cohybridized were combined, dried in a Savant SVC100 Speed-Vac (Farmingdale) and stored at −80°C until hybridization.

Microarrays were constructed as previously described [47]. Briefly, 70-mer probes targeting 2,857 *L. monocytogenes* ORFs were spotted onto Corning UltraGAPS slides (Corning Inc) at the Microarray Core Facility at Cornell University.

Microarray hybridization was performed as described previously [46]. Spotted microarray slides were first incubated for 1 h in a 1% bovine serum albumin-5X SSC-0.1% sodium dodecyl sulfate solution pre-warmed to 42°C. Subsequently, slides were washed twice in 0.1X SSC and twice in filtered water and then dried. The combined cDNA targets were reconstituted in 55 µl hybridization buffer and denatured at 95°C for 5 minutes. Targets were applied to microarray slides and overlaid with mSeries LifterSlips (Erie Scientific) followed by overnight hybridization at 42°C. Slides were then washed 5 minutes in 42°C pre-warmed 2X SSC plus 0.1% SDS, 5 minutes in 2X SCC, and 2.5 min in 0.2X SSC. After a final wash in filtered water, slides were dried and scanned with a GenePix 4000B scanner (Molecular Devices, Sunnyvale, CA).

### Microarray data analysis

The median fluorescence intensity data for all probes on the array were analyzed using LIMMA (linear models for microarray analysis)[48]. The Empirical Bayes method employed in LIMMA is used to borrow information across genes resulting in stable analyses of small samples. This method also allow analysis of datasets when values are missing due to low spot quality parameters [48]. We first normalized the data on each array using print-tip Lowess, followed by log_2_ conversion of the normalized data. A correlation was determined for the signal from duplicate spots of each probe. Significant differences were determined by calculating a moderated t-statistic which is similar to an ordinary t-statistic except that the standard errors have been shrunk towards a common value using a Bayesian model. P-values were calculated for each gene based on the moderated t-statistics and adjusted with the Benjamini-Hochberg false discovery rate correction for multiple tests. Differences in transcripts levels were considered meaningful only when adjusted *P*<0.05 and fold change ≥1.5 fold.

Gene set enrichment analysis (GSEA) [48] was used to identify gene sets that were significantly overrepresented among genes up- or down-regulated in the Δ*lhrA* mutant strain. GSEA was carried out as described in [49].

### Examination of chitinase activity

The chitinase activities of *L. monocytogenes* was measured as previously described [34]. Wild type and mutant strains were spotted on the same chitin agar plate. After incubation, the clearing zone diameter was measured. Pair wise t-test was used for comparisons between the size of the clearing zones of the wild type and each mutant after 4 days of incubation. Zone diameters from ten independent experiments were compared.

## Supporting Information

Figure S1 **Verification of microarray data by qRT-PCR. See Table S1 and Table S2 for more details on the genes tested.**
(TIF)

Table S1 **Genes positively affected by LhrA.**
(DOCX)

Table S2 **Genes negatively affected by LhrA.**
(DOCX)

Table S3 **Primers and TaqMan probes used in this study.**
(DOCX)
