# Supplementary figures and images for: A Small RNA Controls Expression of the Chitinase ChiA in Listeria monocytogenes
Source: PLoS One. 2011 Apr 18;6(4):e19019. doi: 10.1371/journal.pone.0019019 (PMC3078929; doi:10.1371/journal.pone.0019019)

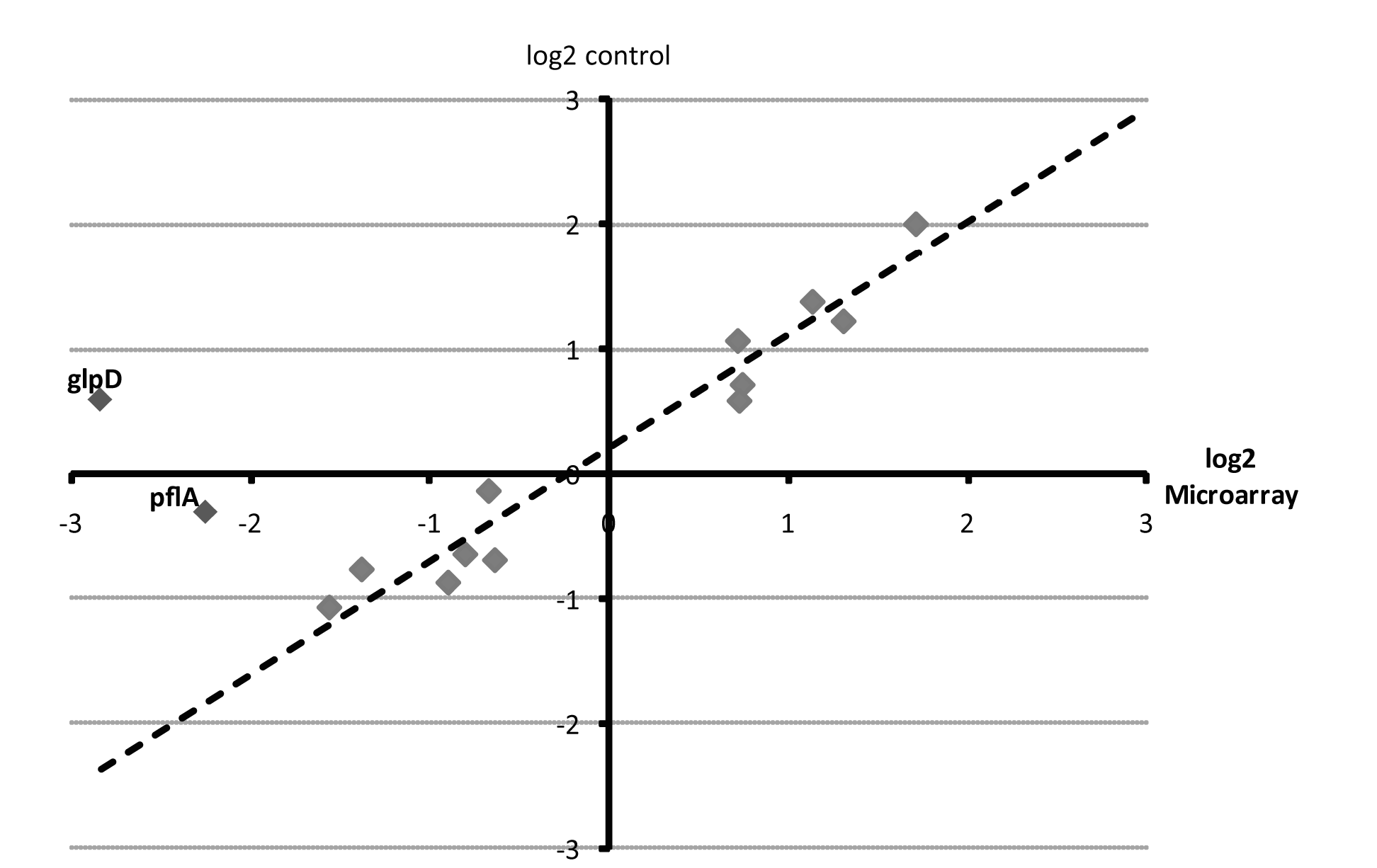

Supplement: Figure S1 — Verification of microarray data by qRT-PCR. See Table S1 and Table S2 for more details on the genes tested. (TIF) [file pone.0019019.s001.tif]
