# Supplementary material for: A Small RNA Controls Expression of the Chitinase ChiA in Listeria monocytogenes
Source: PLoS One. 2011 Apr 18;6(4):e19019. doi: 10.1371/journal.pone.0019019 (PMC3078929; doi:10.1371/journal.pone.0019019)
Supplement: Table S1 — Genes positively affected by LhrA. (DOCX) [file pone.0019019.s002.docx]

**Table S1.** *Genes positively affected by LhrA*.

| ***Gene*** | **Δ*lhrA*/wt ^a^** | **Verification ^b, c^** | **Δ*sigB*/wt ^d^** | **Description** |
| --- | --- | --- | --- | --- |
| *Amino acid biosynthesis* | | | | |
| *lmo0490* | -1.7 |  |  | similar to shikimate 5-dehydrogenase |
| *glnA* | -2.2 |  |  | highly similar to glutamine synthetases |
| *ilvN* | -1.5 |  | Up | similar to acetolactate synthase (acetohydroxy-acid synthase) (small subunit) |
| *alsS* | -2.1 |  | Up | similar to alpha-acetolactate synthase protein, AlsS |
| *lmo2524* | -1.7 |  | Up | similar to hydroxymyristoyl-(acyl carrier protein) dehydratase |
| *thrB* | -1.9 |  | Up | highly similar to homoserine kinase |
| *thrC* | -1.6 |  | Up | highly similar to threonine synthase |
| *Biosynthesis of cofactors, prosthetic groups, and carriers* | | | | |
| *folC* | -1.5 |  | Up | similar to Folyl-polyglutamate synthetase |
| *lmo1592* | -1.9 |  | Up | similar to thiamin biosynthesis protein ThiI |
| *lmo2101* | -2.3 |  | Up | similar to a protein required for pyridoxine synthesis |
| *hemH* | -1.5 |  |  | similar to ferrochelatase |
| *lmo2542* | -1.5 |  |  | similar to protoporphyrinogen oxidase |
| *Cell envelope* | | | | |
| *lmo0441* | -1.5 |  | Up | similar to penicillin-binding protein (D-alanyl-D-alanine carboxypeptidase) |
| *murF* | -1.5 |  | Up | UDP-N-acetylmuramoylalanyl-D-glutamyl-2,6-diamino pimelate-D-alanyl-D-alanyl ligase |
| *dltB* | -1.9 |  | Up | DltB protein for D-alanine esterification of lipoteichoic acid and wall teichoic acid |
| *dltC* | -1.7 |  | Up | D-alanyl carrier protein |
| *dltD* | -2.2 |  | Up | DltD protein for D-alanine esterification of lipoteichoic acid and wall teichoic acid |
| *mreD* | -1.7 |  | Up | similar to cell-shape determining protein MreD |
| *lmo2637* | -2.9 |  | Up | conserved lipoprotein |
| *lmo2691* | -2.0 |  | Up | similar to autolysin, N-acetylmuramidase |
| *Cellular processes* | | | | |
| *lmo0252* | -1.5 |  |  | similar to repressor (penicilinase repressor) |
| *ftsX* | -1.5 |  |  | highly similar to cell-division protein FtsX |
| *Central intermediary metabolism* | | | | |
| *ackA* | -1.7 |  | Down | highly similar to acetate kinase |
| *lmo1736* | -1.5 |  | Up | similar to unknown proteins |
| *lmo2540* | -1.5 |  |  | similar to phosphatases |
| *lmo2638* | -2.3 |  |  | similar to NADH dehydrogenase |
| *lmo2761* | -1.5 |  |  | similar to beta-glucosidase |
| *lmo2800* | -1.6 |  |  | similar to dehydrogenase |
| *DNA metabolism* | | | | |
| *dnaA* | -1.5 |  |  | Chromosomal replication initiation protein DnaA |
| *dnaN* | -2.1 |  |  | DNA polymerase III, beta chain |
| *gyrA* | -1.6 |  | Up | DNA gyrase subunit A |
| *Ssb* | -1.5 |  |  | highly similar to single-strand binding protein (SSB) |
| *lmo2050* | -1.5 |  |  | similar to excinuclease ABC (subunit A) |
| *lmo2676* | -1.6 |  | Up | similar to UV-damage repair protein |
| *lmo2719* | -1.5 |  |  | conserved hypothetical proteins |
| *Energy metabolism* | | | | |
| *lmo0031* | -1.5 |  | Up | transcriptional regulator LacI family |
| *lmo0096* | -1.5 |  |  | similar to PTS system mannose-specific, factor IIAB |
| *lmo0097* | -1.6 |  |  | similar to PTS system mannose-specific, factor IIC |
| *lmo0098* | -1.5 |  | Down | similar to PTS system mannose-specific, factor IID |
| *Ldh* | -2.0 |  | Down | similar to L-lactate dehydrogenase |
| *lmo0734* | -1.7 |  |  | similar to transcriptional regulator (LacI family) |
| *lmo0735* | -1.6 |  |  | similar to Ribulose-5-Phosphate 3-Epimerase |
| *lmo0736* | -1.8 |  |  | similar to ribose 5-phosphate isomerase |
| *lmo0737* | -1.8 |  |  | unknown |
| *lmo0738* | -2.2 |  |  | Phosphotransferase system (PTS) beta-glucoside-specific enzyme IIABC component |
| *lmo0739* | -2.3 |  |  | similar to 6-phospho-beta-glucosidase |
| *glpD* | -7.1 | 1.5 ^b^ | Up | similar to glycerol 3 phosphate dehydrogenase |
| *pflB* | -3.5 |  | Up | pyruvate formate-lyase |
| *lmo1634* | -2.2 |  |  | similar to Alcohol-acetaldehyde dehydrogenase |
| *pflA* | -4.8 | -1.2 ^b^ | Up | similar to pyruvate formate-lyase |
| *gpsA* | -1.5 |  | Up | similar to NAD(P)H-dependent glycerol-3-phosphate dehydrogenase |
| *lmo1992* | -2.9 |  | Down | similar to alpha-acetolactate decarboxylase |
| *lmo2152* | -1.5 |  | Up | similar to thioredoxin |
| *Gap* | -1.8 |  | Down | highly similar to glyceraldehyde 3-phosphate dehydrogenase |
| *atpE* | -1.5 |  |  | highly similar to H+-transporting ATP synthase chain c |
| *fbaA* | -2.0 |  | Up | similar to fructose-1,6-bisphosphate aldolase |
| *lmo2659* | -1.5 |  |  | similar to ribulose-phosphate 3-epimerase |
| *cydB* | -2.2 |  | Up | highly similar to cytochrome D ubiquinol oxidase subunit II |
| *cydA* | -1.5 |  | Up | highly similar to cytochrome D ubiquinol oxidase subunit I |
| *lmo2762* | -1.5 |  |  | similar to PTS cellobiose-specific enzyme IIB |
| *lmo2800* | -1.6 |  |  | similar to dehydrogenase |
| *Fatty acid and phospholipid metabolism* | | | | |
| *lmo0786* | -1.9 |  | Up | similar to acyl-carrier protein phosphodiesterase and to NAD(P)H dehydrogenase |
| *Hypothetical proteins* | | | | |
| *lmo1326* | -1.6 |  | Up | conserved hypothetical protein similar to B. subtilis YlxP protein |
| *lmo1333* | -1.5 |  | Up | similar to B. subtilis YqzC protein |
| *lmo1717* | -1.5 |  |  | similar to hypothetical proteins |
| *lmo1750* | -1.9 |  |  | similar to unknown protein |
| *lmo2048* | -1.5 |  | Up | similar to unknown proteins |
| *lmo2113* | -1.5 |  | Down | similar to unknown proteins |
| *lmo2149* | -1.7 |  | Up | similar to unknown proteins |
| *lmo2151* | -1.5 |  |  | similar to unknown proteins |
| *lmo2187* | -1.6 |  |  | unknown |
| *lmo2669* | -1.5 |  |  | unknown |
| *lmo2675* | -1.6 |  |  | unknown |
| *lmo2767* | -1.9 |  |  | unknown |
| *Mobile and extrachromosomal element functions* | | | | |
| *lmo0152* | -2.0 |  |  | similar to oligopeptide ABC transporter-binding protein |
| *lmo1097* | -1.6 |  |  | similar to integrases |
| *lmo2569* | -1.8 |  | Up | similar to dipeptide ABC transporter (dipeptide-binding protein) |
| *lmo0049* | -1.8 |  |  | unknown |
| *lmo0104* | -1.5 |  | Up | unknown |
| *lmo0412* | -1.8 |  |  | unknown |
| *lmo0451* | -1.5 |  |  | unknown |
| *lmo0471* | -1.5 |  |  | unknown |
| *lmo1188* | -1.5 |  | Up | unknown |
| *lmo1257* | -2.9 | -2.1 ^c^ |  | unknown |
| *lmo1306* | -1.5 |  | Up | highly similar to B. subtilis YneF protein |
| *rpmG* | -1.5 |  |  | ribosomal protein L33 |
| *lmo1649* | -1.5 |  |  | unknown |
| *lmo2129* | -1.6 |  |  | unknown |
| *lmo2156* | -1.5 |  |  | unknown |
| *lmo2642* | -1.6 |  |  | unknown |
| *lmo2828* | -1.8 |  |  | unknown |
| *Protein fate* |  |  |  |  |
| *lmo0960* | -1.6 |  | Up | similar to proteases |
| *lmo0961* | -1.7 |  | Up | similar to proteases |
| *tig* | -1.5 |  |  | trigger factor (prolyl isomerase) |
| *pflC* | -3.1 |  | Up | pyruvate-formate lyase activating enzyme |
| *lmo1603* | -1.5 |  |  | similar to aminopeptidase |
| *clpP* | -1.6 | -1.1 |  | ATP-dependent Clp protease proteolytic subunit |
| *gtcA* | -1.6 |  |  | wall teichoic acid glycosylation protein GtcA |
| *Protein synthesis* | | | | |
| *rpsR* | -1.5 |  | Up | ribosomal protein S18 |
| *lysS* | -1.5 |  | Up | lysyl-tRNA synthetase |
| *rplK* | -1.6 |  | Up | ribosomal protein L11 |
| *rplA* | -1.6 |  |  | ribosomal protein L1 |
| *rplJ* | -2.6 | -1.7 ^c^ | Up | ribosomal protein L10 |
| *rplL* | -2.7 |  | Up | ribosomal protein L12 |
| *rpsT* | -1.8 |  | Up | ribosomal protein S20 |
| *valS* | -1.5 |  |  | valyl-tRNA synthetase |
| *rpsD* | -1.5 |  |  | ribosomal protein S4 |
| *gatB* | -1.6 |  |  | glutamyl-tRNA(Gln) amidotransferase (subunit B) |
| *rplT* | -1.8 |  | Up | ribosomal protein L20 |
| *rpmI* | -1.5 |  | Up | ribosomal protein L35 |
| *rplS* | -1.9 |  | Up | ribosomal protein L19 |
| *rpsP* | -1.6 |  | Up | ribosomal protein S16 |
| *rpmF* | -2.3 |  | Up | ribosomal protein L32 |
| *lmo2448* | -1.5 |  | Up | conserved hypothetical protein |
| *rpmE* | -1.9 |  | Up | ribosomal protein L31 |
| *rpsI* | -1.9 |  | Up | ribosomal protein S9 |
| *rplM* | -1.5 |  | Up | ribosomal protein L13 |
| *rplQ* | -1.7 |  |  | ribosomal protein L17 |
| *rpsE* | -1.5 |  |  | ribosomal protein S5 |
| *rplR* | -1.6 |  | Up | ribosomal protein L18 |
| *rplF* | -1.7 |  | Up | ribosomal protein L6 |
| *rpsH* | -1.8 |  | Up | ribosomal protein S8 |
| *rpsN* | -1.9 |  | Up | ribosomal protein S14 |
| *rplE* | -1.7 |  | Up | ribosomal protein L5 |
| *rplX* | -1.8 |  | Up | ribosomal protein L24 |
| *rplN* | -1.8 |  | Up | ribosomal protein L14 |
| *rpsQ* | -2.1 |  | Up | ribosomal protein S17 |
| *rpmC* | -1.9 |  | Up | ribosomal protein L29 |
| *rplP* | -2.2 |  | Up | ribosomal protein L16 |
| *rpsC* | -2.0 |  | Up | ribosomal protein S3 |
| *rplV* | -2.1 |  | Up | ribosomal protein L22 |
| *rpsS* | -2.0 |  | Up | ribosomal protein S19 |
| *rplB* | -1.7 |  | Up | ribosomal protein L2 |
| *rplW* | -1.8 |  | Up | ribosomal protein L23 |
| *rplC* | -1.7 |  | Up | ribosomal protein L3 |
| *Purines, pyrimidines, nucleosides, and nucleotides* | | | | |
| *prs* | -1.6 |  | Up | phosphoribosyl pyrophosphate synthetase |
| *lmo0280* | -1.7 |  |  | highly similar to anaerobic ribonucleotide reductase activator protein |
| *lmo1827* | -1.5 |  |  | similar to guanylate kinases |
| *pnp* | -1.6 |  | Up | similar to purine-nucleoside phosphorylase |
| *pdp* | -1.7 |  | Up | similar to pyrimidine-nucleoside phosphorylase |
| *pyrG* | -1.6 |  |  | highly similar to CTP synthases |
| *Regulatory functions* | | | | |
| *lmo0048* | -1.5 |  |  | similar to Staphylococcus two-component sensor histidine kinase AgrB |
| *lmo0051* | -1.5 |  |  | similar to 2-components response regulator protein (AgrA from Staphylococcus) |
| *lmo0161* | -1.5 |  |  | unknown |
| *lmo0402* | -1.8 |  | Down | similar to transcriptional antiterminator (BglG family) |
| *lmo0725* | -1.5 |  | Up | putative peptidoglycan bound protein (LPXTG motif) |
| *lmo0788* | -2.1 |  | Down | unknown |
| *lmo0797* | -1.5 |  | Up | unknown |
| *lmo1172* | -1.3 |  |  | similar to similar to two-component response regulator |
| *lmo1173* | -1.8 |  | Up | similar to two-component sensor histidine kinase |
| *glnR* | -1.9 | -1.8 ^b^ | Up | similar to glutamine synthetase repressor |
| *lmo2173* | -2.1 |  |  | similar to sigma-54-dependent transcriptional activator |
| *lmo2179* | -1.9 |  |  | putative peptidoglycan bound protein (LPXTG motif) |
| *lmo2764* | -1.8 |  |  | similar to xylose operon regulatory protein and to glucose kinase |
| *lmo2792* | -1.6 |  |  | unknown |
| *Signal transduction* | | | | |
| *lmo2763* | -1.9 |  |  | similar to PTS cellobiose-specific enzyme IIC |
| *Transport and binding proteins* | | | | |
| *lmo0179* | -1.7 |  | Up | similar to sugar ABC transporters, permease proteins |
| *lmo0181* | -1.5 |  |  | similar to sugar ABC transporter, sugar-binding protein |
| *lmo0283* | -1.6 |  | Up | similar to ABC transporter permease protein |
| *lmo0284* | -1.6 |  |  | similar to ABC transporter (ATP-binding protein) |
| *lmo0947* | -1.5 |  |  | hypothetical transport protein |
| *clpE* | -1.9 |  |  | ATP-dependent protease |
| *lmo1003* | -1.5 |  |  | phosphotransferase system enzyme I |
| *lmo1224* | -1.5 |  |  | similar to different proteins |
| *lmo1389* | -1.5 |  |  | similar to sugar ABC transporter, ATP-binding protein |
| *lmo1390* | -1.6 |  |  | similar to ABC transporter (permease proteins) |
| *lmo1391* | -1.8 |  |  | similar to sugar ABC transporter, permease protein |
| *lmo1746* | -1.5 |  |  | similar to ABC transporter (permease) |
| *lmo1997* | -1.5 |  |  | similar to PTS mannose-specific enzyme IIA component |
| *lmo2192* | -1.5 |  |  | similar to oligopeptide ABC transporter (ATP-binding protein) |
| *cydD* | -1.6 |  |  | ABC transporter (ATP-binding protein) required for expression of cytochrome BD |
| *cydC* | -1.5 |  |  | highly similar to ABC transporter required for expression of cytochrome BD |
| *lmo2765* | -1.5 |  |  | similar to PTS cellobiose-specific enzyme IIA |
| *lmo2799* | -1.7 |  |  | similar to phosphotransferase system mannitol-specific enzyme IIBC |
| *Unclassified* | | | | |
| *lmo0130* | -1.7 |  |  | similar to 5-nucleotidase, putative peptidoglycan bound protein (LPXTG motif) |
| *hly* | -1.8 | -1.6 ^b^ |  | listeriolysin O precursor |
| *lmo0285* | -1.6 |  |  | putative lipoprotein |
| *lmo0354* | -2.0 |  |  | similar to fatty-acid--CoA ligase |
| *lmo0355* | -2.5 |  | Up | similar to Flavocytochrome C Fumarate Reductase chain A |
| *lmo0394* | -1.6 |  |  | similar to L. monocytogenes extracellular P60 protein |
| *lmo0814* | -1.6 |  |  | similar to oxidoreductases |
| *lmo0912* | -1.5 |  |  | similar to transporters (formate) |
| *dltA* | -1.6 | -1.6 ^b^ |  | D-alanine-activating enzyme (dae), D-alanine-D-alanyl carrier protein ligase (dcl) |
| *lmo2029* | -1.6 |  |  | similar to unknown proteins |
| *lmo2102* | -2.2 |  |  | unknown |
| *lmo2142* | -1.8 |  |  | unknown |
| *lmo2269* | -2.7 |  | Down | unknown |
| *lmo0624* | -1.7 |  |  | similar to unknown proteins |
| *lemA* | -1.6 |  | Up | Listeria epitope LemA |
| *Viral functions* | | | | |
| *lmo2504* | -1.5 |  |  | similar to cell wall binding proteins |
| *spl* | -1.7 |  |  | peptidoglycan lytic protein P45 |

^a^ Fold of change

^b^ Verified by quantitative RT-PCR

^c^ Verified by Northern blotting

^d^ Genes regulated by σ^B^ after NaCl stress or in stationary growth phase, as described by Raengpradub et al. [1]

Reference List

1. Raengpradub S, Wiedmann M, Boor KJ (2008) Comparative analysis of the sigma B-dependent stress responses in Listeria monocytogenes and Listeria innocua strains exposed to selected stress conditions. Appl Environ Microbiol 74: 158-171. AEM.00951-07 [pii];10.1128/AEM.00951-07 [doi].
