# Supplementary material for: A Small RNA Controls Expression of the Chitinase ChiA in Listeria monocytogenes
Source: PLoS One. 2011 Apr 18;6(4):e19019. doi: 10.1371/journal.pone.0019019 (PMC3078929; doi:10.1371/journal.pone.0019019)
Supplement: Table S2 — Genes negatively affected by LhrA. (DOCX) [file pone.0019019.s003.docx]

**Table S2.** Genes negatively affected by LhrA.

| **Name** | **Δ*lhrA*/wt ^a^** | **Verification ^b, c^** | **Δ*sigB*/wt ^d^** | **Description** |
| --- | --- | --- | --- | --- |
| *Unknown function* | | | | |
| *lmo0590* | 2.0 |  | Down | similar to a fusion of two types of conserved hypothetical proteinconserved hypothetical |
| *lmo0591* | 2.0 |  | Down | similar to unknown membrane proteins |
| *lmo0755* | 1.6 |  |  | similar to unknown proteins |
| *lmo1576* | 2.1 |  | Down | similar to unknown proteins |
| *lmo1622* | 1.6 |  | Down | similar to unknown proteins |
| *lmo2053* | 1.5 |  |  | similar to unknown proteins |
| *lmo2403* | 1.6 |  |  | similar to B. subtilis YunD protein |
| *Transport and binding proteins* | | | | |
| *lmo0667* | 1.9 |  |  | similar to ABC transporter (ATP-binding protein) |
| *lmo0782* | 1.7 |  | Down | similar to mannose-specific phosphotransferase system (PTS) component IIC |
| *lmo0783* | 2.1 |  | Down | similar to mannose-specific phosphotransferase system (PTS) component IIB |
| *lmo0784* | 1.7 | 1.6 ^b^ | Down | similar to mannose-specific phosphotransferase system (PTS) component IIA |
| *lmo1372* | 1.6 |  | Down | similar to branched-chain alpha-keto acid dehydrogenase E1 subunit |
| *opuCA* | 1.5 | 1.2 ^b^ | Down | similar to glycine betaine/carnitine/choline ABC transporter (ATP-binding protein) |
| *lmo1889* | 1.6 |  |  | similar to conserved hypothetical proteins |
| *Transcription* | | | | |
| *lmo1246* | 1.8 |  |  | similar to ATP-dependent RNA helicase (DEAD motif) |
| *Regulatory functions* | | | | |
| *lmo0612* | 1.5 |  |  | similar to transcription regulator MarR family |
| *lmo0902* | 1.7 |  |  | similar to transcription regulator (GntR family) |
| *lmo1878* | 1.5 |  | Down | similar o transcriptional regulators |
| *lmo2085* | 1.8 |  | Down | putative peptidoglycan bound protein (LPXTG motif) |
| *lmo2366* | 1.6 |  | Down | similar to transcription regulator DeoR family |
| *lmo2493* | 1.7 |  |  | similar to transcription regulator ArsR family |
| *Protein synthesis* | | | | |
| *lmo2076* | 1.6 |  |  | similar to ribosomal protein alanine acetyltransferase |
| *Protein fate* | | | | |
| *lmo1269* | 1.6 |  | Down | similar to type-I signal peptidase |
| *lmo1666* | 1.5 |  | Down | peptidoglycan linked protein (LPxTG) |
| *lmo0302* | 2.5 | 2.3 ^b, c^ | Down | unknown |
| *lmo0303* | 2.2 | 2.5 ^c^ |  | putaive secreted, lysin rich protein |
| *lmo0310* | 1.5 |  | Down | unknown |
| *lmo0665* | 1.5 |  |  | unknown |
| *lmo0777* | 1.5 |  | Up | unknown |
| *lmo1121* | 1.7 |  |  | unknown |
| *lmo1123* | 1.8 |  |  | unknown |
| *lmo2258* | 3.4 |  | Up | unknown |
| *lmo2492* | 1.6 |  | Down | unknown |
| *Hypothetical proteins* | | | | |
| *lmo0133* | 1.5 |  | Down | similar to E. coli YjdI protein |
| *lmo0589* | 1.9 |  | Down | unknown |
| *lmo0592* | 1.6 |  | Down | unknown |
| *lmo0596* | 1.7 | 1.5 ^b^ | Down | similar to unknown proteins |
| *lmo0794* | 1.7 |  | Down | similar to B. subtilis YwnB protein |
| *lmo0951* | 1.7 |  |  | unknown |
| *lmo0995* | 1.5 |  | Down | similar to B. subtilis YkrP protein |
| *lmo1058* | 1.6 |  |  | similar to B. subtilis YktA protein |
| *lmo1124* | 1.8 |  |  | unknown |
| *lmo1125* | 1.5 |  |  | unknown |
| *lmo1243* | 1.7 |  | Down | unknown |
| *lmo1528* | 1.5 |  |  | similar to unknown proteins |
| *lmo1535* | 1.8 |  | Down | similar to unknown proteins |
| *lmo1612* | 1.5 |  | Up | similar to unknown proteins |
| *lmo1890* | 1.6 |  |  | similar to conserved hypothetical proteins |
| *lmo2042* | 1.6 |  | Down | similar to unknown proteins |
| *lmo2061* | 1.6 |  |  | similar to unknown protein |
| *lmo2078* | 1.6 |  |  | similar to unknown proteins |
| *lmo2402* | 1.6 |  |  | similar to B. subtilis YutD protein |
| *lmo2527* | 1.5 |  | Down | similar to B. subtilis YwzB protein |
| *Energy metabolism* | | | | |
| *qoxA* | 1.6 |  | Down | AA3-600 quinol oxidase subunit II |
| *lmo0305* | 1.6 |  | Down | similar to low specificity L-allo-threonine aldolase |
| *lmo0411* | 2.0 |  | Down | similar to phosphoenolpyruvate synthase (N-terminal part) |
| *lmo0722* | 1.5 |  | Down | similar to pyruvate oxidase |
| *lmo0811* | 1.6 |  | Down | similar to carbonic anhydrase |
| *lmo0913* | 1.7 |  | Down | similar to succinate semialdehyde dehydrogenase |
| *trxA* | 1.6 |  |  | thioredoxin |
| *lmo1371* | 1.8 |  | Down | similar to branched-chain alpha-keto acid dehydrogenase E3 subunit |
| *lmo1373* | 1.6 |  | Down | similar to branched-chain alpha-keto acid dehydrogenase E1 subunit |
| *lmo1534* | 1.5 |  | Down | similar to L-lactate dehydrogenase |
| *lmo1883* | 3.3 | 5.7 ^c^ | Down | similar to chitinases |
| *lmo2467* | 1.6 |  |  | similar to chitinase and chitin binding protein |
| *trxB* | 1.6 |  |  | thioredoxin reductase |
| *lmo2712* | 1.6 |  |  | highly similar to gluconate kinase |
| *lmo2783* | 1.9 |  |  | similar to cellobiose phosphotransferase system enzyme IIC |
| *DNA metabolism* | | | | |
| *ruvB* | 1.5 |  |  | highly similar to Holliday junction DNA helicase RuvB |
| *ruvA* | 1.7 |  |  | highly similar to Holliday junction DNA helicase (ruvA) |
| *lmo1621* | 1.6 |  |  | weakly similar to E. coli MutT protein (dGTP pyrophosphohydrolase |
| *recU* | 1.6 |  |  | similar to DNA repair and homologous recombination protein |
| *Central intermediary metabolism* | | | | |
| *lmo0663* | 1.5 |  |  | conserved hypothetical proteins |
| *lmo1684* | 1.7 |  |  | similar to glycerate dehydrogenases |
| *lmo2390* | 1.5 |  | Down | similar to hypothetical thioredoxine reductase |
| *lmo2453* | 1.8 |  | Down | similar to lipolytic enzyme |
| *lmo2573* | 1.6 |  | Down | similar to zinc-binding dehydrogenase |
| *Cellular processes* | | | | |
| *lmo0291* | 1.5 |  | Down | conserved hypothetical protein similar to B. subtilis YycJ protein |
| *lmo0669* | 1.7 | 2.1 ^b^ | Down | similar to oxidoreductase |
| *lmo1577* | 1.8 |  |  | similar to unknown proteins |
| *lmo1694* | 1.8 |  | Down | similar to CDP-abequose synthase |
| *lmo1888* | 1.5 |  |  | similar to hypothetical proteins |
| *lmo2084* | 1.7 |  |  | unknown |
| *lmo2230* | 1.5 |  | Down | similar to arsenate reductase |
| *lmo2755* | 1.6 |  | Down | similar to acylase and diesterase |
| *Cell envelope* | | | | |
| *lmo0880* | 2.0 |  | Down | similar to wall associated protein precursor (LPXTG motif) |
| *pbpA* | 1.5 |  |  | similar to penicillin-binding protein 2A |
| *lmo2590* | 1.6 |  |  | similar to ATP binding proteins |
| *Biosynthesis of cofactors, prosthetic groups, and carriers* | | | | |
| *lmo0629* | 1.5 |  | Down | unknown |
| *lmo1042* | 1.7 |  | Down | similar to molybdopterin biosynthesis protein moeA |
| *lmo1043* | 1.6 |  | Down | similar to molybdopterin-guanine dinucleotide biosynthesis MobB |
| *lmo1045* | 1.6 |  | Down | similar to molybdopterin converting factor (subunit 1). |
| *Amino acid biosynthesis* | | | | |
| *argB* | 1.7 |  |  | highly similar to N-acetylglutamate 5-phosphotransferase |

^a^ Fold of change

^b^ Verified by quantitative RT-PCR

^c^ Verified by Northern blotting

^d^ Genes regulated by σ^B^ after NaCl stress or in stationary growth phase, as described by Raengpradub et al. [1]

Reference List

1. Raengpradub S, Wiedmann M, Boor KJ (2008) Comparative analysis of the sigma B-dependent stress responses in Listeria monocytogenes and Listeria innocua strains exposed to selected stress conditions. Appl Environ Microbiol 74: 158-171. AEM.00951-07 [pii];10.1128/AEM.00951-07 [doi].
