# Supplementary material for: A Small RNA Controls Expression of the Chitinase ChiA in Listeria monocytogenes
Source: PLoS One. 2011 Apr 18;6(4):e19019. doi: 10.1371/journal.pone.0019019 (PMC3078929; doi:10.1371/journal.pone.0019019)
Supplement: Table S3 — Primers and TaqMan probes used in this study. (DOCX) [file pone.0019019.s004.docx]

**Table S3.** Primers used in this study.

| Name | Sequence (5´-3´) | Description |
| --- | --- | --- |
| LhrA-29 | GGGGAATTCTTTTCAAGAACAATAGTAAAATAAGTTATAGG^a^ | Forward primer, plhrA29-lacZ |
| LhrA-36 | GGGGAATTCTTGCTTTTTTCAAGAACAATAGTAAAATAAG^a^ | Forward primer, plhrA36-lacZ |
| LhrA-61 | GGGGAATTCGTCACAAAAAATCTATATTTATTTAC^a^ | Forward primer, plhrA61-lacZ |
| LhrA-83 | GGGGGAATTCAATTCTCATCTTTCTTATCTTCG^a^ | Forward primer, plhrA83-lacZ |
| LhrA-157 | GGGGGAATTCGGGGACCTTTAATGGTTTCC^a^ | Forward primer, plhrA157-lacZ |
| sRNA1_13 | CCCCGGATCCTGATAGGGTCTTATTCCCATG^b^ | Reverse primer, plhrA-lacZ fusions |
| Lmo0302-F | GGGGGAATTCCTTCTTTCAGTGCCTCAAAAGCA^a^ | Forward primer, *lmo0302*-*lacZ* fusion |
| Lmo0302-R | GGGGGGATCCCATCCGTGTACTGTTTTTTTCATC^b^ | Reverse primer, *lmo0302*-*lacZ* fusion |
| Lmo0669-F | GGGGGAATTCTCGATGTTACCGCCAATCTGG^a^ | Forward primer, *lmo0669-lacZ* fusion |
| Lmo0669-R | GGGGGGATCCACTTTTTTATTTCCCATTTTCATCCC^b^ | Reverse primer, *lmo0669-lacZ* fusion |
| Lmo0880-F | GGGGGAATTCTGAGCGGTTTTGAGCTAGAAG^a^ | Forward primer, *lmo0880-lacZ* fusion |
| Lmo0880-R | GGGGGGATCCAGCCATCTTTTTTTCATTTTGTTTCAT^b^ | Reverse primer, *lmo0880-lacZ* fusion |
| V-lac2 | CTTCCACAGTAGTTACACCACC | Primer extension, lacZ-fusions |
| lmo0784-F | CGGCAGTCCTTATAACGCATCT | Lmo0784 RT-PCR |
| lmo0784-R | CCCCCGTTACAACATCCATATT | Lmo0784RT-PCR |
| lmo0784P | TCAAATCGCCCTTCCAGA | Lmo0784TaqMan probe |
| dltA-1170F | TGGTATTATCAAAGACGGTTATCTGTTC | dltA RT-PCR |
| dltA-1247R | TCGATGCGGTAACCATGAAG | dltA RT-PCR |
| dltAP | CCAAGGACGTCTTGACTT | dltA TaqMan probe |
| glnR-87F | TCGCTACTATGAAGACCAAGGATTAAT | glnR RT-PCR |
| glnR-162F | GTCTTGAAGTGAATACAAACGGTGATT | glnR RT-PCR |
| glnRp | CATCCGGCACGAA | glnR TaqMan probe |
| lmo0302-128F | TGAATTTTGGTGATGATGGAGTAGA | Lmo0302 RT-PCR |
| lmo0302-203R | TTATCCAGCGAAACTCGAACACT | Lmo0302 RT-PCR |
| lmo0302P | CTCTTTCGCATTTAACA | Lmo0302 TaqMan probe |
| Lm669TqMnF1 | tcaagctatcaaggcgctaataaa | Lmo0669 RT-PCR |
| Lm669TqMnR1 | ccgaccaattccggagtct | Lmo0669 RT-PCR |
| Lmo0669P | AACCGGGAAAAAAGCCTTTGTAACAGGG | Lmo0669 TaqMan probe |
| pflA-772F | AACGGTGCTGCAAGCTCATT | pflA RT-PCR |
| pflA-835R | ATCGCGTTGGATGTAGATATCAAG | pflA RT-PCR |
| pflAP | CGTATTCCGATTTTC | pflA TaqMan probe |
| glpD-145F | CTTGTTGAAATGGGTGATTTCG | glpD RT-PCR |
| glpD-222R | TAAATATCTTAATCCACCGTGGACTAATT | glpD RT-PCR |
| glpDP | CATCGAGCCGTTCAA | glpD TaqMan probe |
| hly TqMnF | atggcaccaccagcatctc | hly RT-PCR |
| hly TqMnR | atccgcgtgtttcttttcgat | hly RT-PCR |
| hlyP | CCTGCAAGTCCTAAGAC | hly TaqMan probe |
| lmo0596F | TTCTGGTATTTTCCACACAATCTCTT | Lmo0596 RT-PCR |
| lmo0596R | GTCAGCTAGTACCCAGCCAGAAA | Lmo0596 RT-PCR |
| Lmo0596P | TTTTCAGACAGAAAATCACAAA | Lmo0596 TaqMan probe |
| LhrA3 | ttgccatcatgttcgggc | LhrA Northern blot probe |
| lmo0302 NB | TCCGCACCTTCTTATCCAGCGAAACTCGAA | Lmo0302 Northern blot probe |
| lmo0303 NB | TCCCCGACGATTCCAACTTCCATTTTCCGG | Lmo0303 Northern blot probe |
| lmo1883 NB | TTTCCGGAAGATTTCCAACTATGCCAGTACCCA | Lmo1883 (chiA) Northern blot probe |
| 5S rRNA_LMO | GAGAAGCTTAACTACCGTGTTCGGGATGGGAACGG | 5S RNA Northern blot probe |
| T7_lmo0302 | GCGCGAATTCTAATACGACTCACTATAGGTTAGAGAGA-AAATATTACATAGGA^c^ | Forward primer, *in vitro* transcription of lmo0302 |
| Lmo0302­_toe-2 | ATACTCGTACGTCTAAACTAGA | Reverse primer, Toeprint |
| T7_lmo1883 | GGGGGAATTCTAATACGACTCACTATAGTAGCTCCAAA  AAAGTAAGTTTGGGGTA^c^ | Forward primer, *in vitro* transcription of *chiA* (lmo1883) |
| Lmo1883­_in vitro_R | GGGGGGATCCTAAAAGTAAAGACAAACCACCAA | Reverse primer, *in vitro* transcription |
| Lmo1883_toe | CCCAGTCCCGCACCAACTAAA | Reverse primer, Toeprint |
| 16S-rRNA-rev | TGCTCCGTCAGACTTTCGTC | 16S Northern blot, reverse primer |
| 16S-rRNA-fwd | GATGCATAGCCGACCTGAGA | 16S Northern blot, forward primer |

^a^ Restriction site for EcoRI is underlined.

^b^ Restriction site for BamHI is underlined.

^c^ T7 promoter is underlined.
